# Supplementary material for: Force-triggered rapid microstructure growth on hydrogel surface for on-demand functions
Source: Nat Commun. 2022 Oct 20;13:6213. doi: 10.1038/s41467-022-34044-8 (PMC9585076; doi:10.1038/s41467-022-34044-8)
Supplement: Supplementary file 1 — Supplementary information [file 41467_2022_34044_MOESM1_ESM.pdf]

Supplementary Information for

Force-triggered rapid microstructure growth on hydrogel surface for on-demand functions

Qifeng Mu[1], Kunpeng Cui[2], Zhi Jian Wang[1], Takahiro Matsuda[3], Wei Cui[3], Hinako Kato[1], Shotaro Namiki[1], Tomoko Yamazaki[1], Martin Frauenlob[1], Takayuki Nonoyama[3], Masumi Tsuda[2,4], Shinya Tanaka[2,4], Tasuku Nakajima[2,3]*, Jian Ping Gong[2,3]*

[1]Graduate School of Life Science, Hokkaido University, N21W11, Kita-ku, Sapporo 001-0021, Japan;

[2]Institute for Chemical Reaction Design and Discovery (WPI-ICReDD), Hokkaido University, N21W10, Kita-ku, Sapporo 001-0021, Japan;

[3]Faculty of Advanced Life Science, Hokkaido University, N21W11, Kita-ku, Sapporo 001-0021, Japan;

[4]Department of Cancer Pathology, Faculty of Medicine, Hokkaido University, N15, W7, Kita-ku, Sapporo 060-8638, Japan

*E-mail: tasuku@sci.hokudai.ac.jp; gong@sci.hokudai.ac.jp

This PDF file includes:

Materials and Methods
Supplementary Text
Supplementary Figure 1 to Figure 29
Supplementary Table 1
Supplementary References

Table of Contents

Ⅰ. Materials

Ⅱ. Surface structure characterization of double-network (DN) hydrogels

Ⅲ. Kinetics of force-triggered radical polymerisation

Ⅳ. Effect of surface layer on surface chemical modification

Ⅴ. Monomer conversion ratio in force-triggered radical polymerisation

Ⅵ. Programmable complex patterning on DN hydrogel surfaces

Ⅶ. Supplementary figures and table

Materials

Water (H_2O) used was purified by a lab water purification system (Merck KGaA, Darmstadt, Germany). Acrylamide (AAm) was purchased from Junsei Chemical, 2-acrylamido-2-methylpropanesulfonic acid sodium salt (NaAMPS) (49.7 wt% aqueous solution) was provided by Toa Gosei. *N,N′*-Methylenebisacrylamide (MBA), 2-oxoglutaric acid (α-keto), *N*-isopropylacrylamide (NIPAm), acrylic acid (AAc), sodium *p*-styrenesulfonate (NaSS), 3-(methacryloylamino)propyltrimethylammonium chloride (MPTC), and *N,N*-dimethylacrylamide (DMA) were purchased from Fujifilm Wako Pure Chemical Corporation. 8-anilino-1-naphthalenesulfonic acid (ANS) was purchased from Tokyo Chemical Industry. Deuterium oxide (D_2O), sodium hydroxide (NaOH), and hydrochloric acid (HCl) were purchased from Fujifilm Wako Pure Chemical Corporation. Monomer NIPAm was recrystallized from the mixture of n-hexane and toluene (2:3, *V/V*) before use. Other chemicals were used as received.

Surface structure characterization of DN hydrogels

Attenuated total reflectance Fourier-transform infrared (ATR FT-IR) spectroscopy measurement. The hydrogel surface layer chemical signal was probed by an ATR FT-IR spectroscopy (FT-IR 6600 spectrometer, JASCO, Japan). With its shallow detection range (1–2 μm) and high resolution in wavenumber (~2 cm^{-1}), the ATR FT-IR measurements focused on the chemical group at the interface between the hydrogel and its immediate surroundings. We confirmed that the first brittle network PNaAMPS is on the DN hydrogel surfaces based on the stretching vibration peak (1042 cm^{-1}) of sulfonic group from PNaAMPS chains[1]. The as-prepared double-network (DN) and single-network (SN) hydrogels were immersed in pure water for at least one week to equilibrium swelling then immersed in D_2O for two days prior to the measurements. The wavenumber region was 1800–800 cm^{-1}, number of scans was 64, resolution was 2 cm^{-1}. The infrared spectra and linear polymer structure with functional groups were shown in Supplementary Figure 2. Infrared absorption peak at 1042 cm^{-1} indicated that there is first network on the tough DN hydrogel surfaces prepared by our surface-bulk transition technology.

Hydrogel surface elastic modulus. The surface local modulus of the hydrogels was characterized by micro-indentation method. Firstly, as-prepared

different hydrogels were cut into fixed square shape (size: 2 cm × 2 cm, thickness: 3.5–5 mm) then immersed in pure water to equilibrium state prior to the indentation measurement. The square samples were fixed on a rigid plate in a home-made box. A sphere metal indenter (radius: ~0.25 mm) was applied to hydrogels at a constant rate of 1 mm min^{-1} under water[2]. The surface elastic modulus E (Pa) was estimated using the Hertzian contact theory by the following equation[3,4]

$$E = \frac{3}{4} l^{-\frac{3}{2}} f R^{-\frac{1}{2}} (1 - v_P^2) \quad (1)$$

where l is depth, f is indentation force, R is the radius of sphere indenter, and v_p is Poisson's ratio and was set to 0.5. The surface elastic modulus was calculated from the initial slope (indentation depth: 0–0.01 mm) of the f-$l^{3/2}$ indentation curves in Supplementary Figure 3. The calculated surface modulus was the average value of five replicates, which was summarized in Supplementary Table 1.

Kinetics of force-triggered radical polymerisation

Time-resolved near-infrared spectroscopy measurements. To study the kinetics of force-triggered radical polymerisation with different monomers, a time-resolved near-infrared spectroscopy was used. The rapid scan system of the spectroscopy enables the instrument to measure the spectrum every 0.07–0.08 s (one scan per one data) for providing real-time analysis of reaction kinetics. The DN hydrogels immersed in 1.0 M monomer solutions of NIPAm or NaAMPS were stretched to a tensile strain (ε = 3, above their yielding point) in a customized small glove box filled with argon, the strain rate was ~3 s^{-1} and it took approximately ~1 s in the loading process, the tensile stress was around ~0.9 MPa, the hydrogels were immediately unloaded after the loading, the thickness of DN hydrogels with different monomers was 1.5–2 mm. Monomer concentration decay during the polymerisation was detected at wavenumber 6175 cm^{-1} (corresponds to C–H overtone stretching at the C=C double bond) by the near-infrared spectroscopy[5] at 25 °C (Supplementary Figure 6). For smoothing the data, we took the moving average of the absorbance with a width of ± 0.3 s.

Effect of surface layer on surface chemical modification

The DN gel-glass (with surface PAAm layer) was immersed into 1.0 M NaAMPS aqueous solution or into pure water, performed mechanical indentation, and immersed in pure water. As shown in Supplementary Figure 15, height of the grown microstructure of the DN gel-glass with NaAMPS is 43 μm, which is significantly higher than that of the DN gel-glass without NaAMPS monomer supply (~20 μm). The higher microstructure of the DN gel-glass with NaAMPS indicates the polymerization of PNaAMPS chains in the hydrogels despite the presence of the surface layer.

As another experiment, the two types of DN hydrogels were immersed into NIPAm monomer solution and performed mechanical indentation in order to synthesize thermo-responsive PNIPAm on the top-most surface. Supplementary Figure 16 shows the contact angle of a water droplet on the two DN hydrogels at different temperatures after the indentation. The DN gel-Si-PET-0.8kPa shows a significant increase of the surface hydrophobicity at elevated temperature, suggesting existence of PNIPAm on the modified surface of the DN gel-Si-PET-0.8kPa. On the contrary, water contact angle of the DN gel-glass was not sensitive to temperature, suggesting there is no PNIPAm on the surface. Thus, we concluded that it is necessary to use a hydrophobic mold and a reduced surface layer for the hydrogel surface chemical modification triggered by mechanical force. Although the radical polymerization in “bulk” DN hydrogel certainly occurs, the thick surface layer covers the newly synthesized polymers.

Monomer conversion ratio in force-triggered radical polymerisation

In-situ transmission Fourier-transform near-infrared (FT-NIR) spectroscopy measurement. The conversion ratio is an indicator of monomer reactivity in radical polymerisation. To quantify different monomers conversion ratio in the force-triggered radical polymerisation, an in-situ transmission FT-

NIR spectroscopy was used. The near-infrared spectroscopy is sensitive to overtones and combinations of vibrations. There was a characteristic absorption peak at approximately 1613–1639 nm (wavenumber is 6200–6100 cm^{-1}, within the near-infrared region) corresponds to C–H overtone stretching at the C=C double bond of monomers[5]. We performed the measurements in a customized small glove box filled with argon, DN hydrogels fed with different monomers were stretched to a tensile strain (ε = 3) by a set of home-made clamps, the strain rate was ~1 s^{-1} and it took approximately ~6 s in the loading-unloading process, the tensile stress was around ~0.9 MPa. The absorbance *A* was divided by sample thickness *l*, i.e., the normalized absorbance *A/l.* The normalized absorbance was analysed by the Beer-Lambert law[6], which was normally combined in the following mathematical equation.

$$A = \log_{10}\left(\frac{I_O}{I}\right) = \epsilon l c \tag{2}$$

$$A/l \propto c \tag{3}$$

where ϵ is the molar absorption coefficient ($M^{-1}cm^{-1}$), *c* is the molar concentration of the attenuating substances (M), I_o is the intensity of incident infrared light, and *I* is the intensity of transmitted infrared light. Note that ϵ is a characteristic constant related to the substances, so the value of *A/l* is proportional to the molar concentration of substances.

The normalized absorbance *A/l* is proportional to monomer concentration *c* based on the Beer-Lambert law. The measurement background was the DN hydrogel without monomers, the baseline of water and polymer networks was subtracted in this experimental section. We firstly confirmed the transmission near-infrared spectrum calibration curves of DN hydrogels fed with different monomers (monomer concentration from 0 M to 1.0 M) prior to the monomer conversion ratio measurements. A linear correlation between the normalized absorbance peak area *S* and the monomer concentration *c* was observed for DN hydrogels fed with different monomers (Supplementary Figure 17). The observed normalized absorption peak area *S* (selected peak area region within 6250–6100 cm^{-1}, unit/cm) depended on the monomer concentration.

$$S = \alpha c \tag{4}$$

Where α is the proportional constant which slightly depends on the monomer species (α is 9.14, 9.30, 11.95, and 10.38 for the monomers NIPAm, AAc, NaSS, and MPTC, respectively).

We measured the change of monomer concentration in tough DN hydrogel

bulks before stretching and after stretching using the in-situ FT-NIR. The force-triggered variation in monomer concentration was associated with a change in transmitted light intensity at the monomer peak position around 6200–6100 cm^{-1}.

Here, the sample thickness *l* was the initial thickness of DN hydrogels with monomers before stretching, under an assumption that thickness change by stretching is negligibly small. Since the normalized absorption peak area *S* of different monomers is proportional to monomer concentration, the monomer conversion ratio (Conv) can be calculated based on the equation.

$$\begin{aligned}\mathrm{Conv} &= (1 - c_{\mathrm{t}}/c_{\mathrm{o}}) \\ &= (1 - S_1/S_0)\end{aligned} \tag{5}$$

Here, c_t and c_0 are the monomer concentration at time *t*, and initial monomer concentration, respectively. S_1 and S_0 are the normalized absorption peak area of the monomers at time *t* and the initial time. The conversion ratio of monomer NIPAm was around 0.7 in the force-triggered radical polymerisation at 1 min after the stretch. Then we measured the transmission near-infrared spectrum change for different monomers (initial concentration was 1.0 M) when the radical polymerisation time was fixed at 1 min, as shown in Supplementary Figure 18. Monomer conversion ratios for different monomers (NaAMPS, AAc, NaSS, and MPTC) were 0.802 ± 0.005, 0.498 ± 0.016, 0.459 ± 0.008, and 0.593 ± 0.025, respectively. Error ranges represent standard deviation from three replicates. The measurement parameters for in-situ transmission FT-NIR were constant in the experiment. The number of scans was 32 that took around 20 s (hence the measured values at 1 min are the average of approximately 60–80 s after the stretching), resolution was 2 cm^{-1}, detector was triglycine sulfate (TGS), region of the spectral scan was 6300–6000 cm^{-1}, sample thickness was around ~2.5 mm.

Programmable complex patterning on DN hydrogel surfaces

Preparation of stamp with embossed patterns. The stamps with various embossed patterns were printed using a commercial high-precision three-dimensional (3D) printer system (Agilista 3100, Keyence Co., Ltd.). The embossed patterns on the stamps were designed by a commercial software Solidworks (Dassault Systèmes SolidWorks Corporation, USA). The material of 3D printed stamps is high-strength resin. The topographic height of patterns is

~2 mm. The photos of 3D printed stamps are shown in Supplementary Figure 24.

Fluorescent imaging of complex patterns formed by force-triggered polymerisation. The force stamping on DN hydrogels was performed in a glove box. Firstly, the 3D printed stamps were fixed on a home-made metal holder, and the holder was fixed on the sensor of a small tensile machine (MCT-2150, A&D Co., Ltd.) using a screw. Prior to stamping, the DN hydrogels were immersed in NIPAm-ANS aqueous solution (NIPAm: 1.0 M, ANS: 0.3 mM) for overnight. The immersed DN hydrogels were fixed on a rigid glass plate by glue, then were moved to an argon glove box to remove oxygen at least two hours. The stamping depth was around 1 mm. After the force stamping, the DN hydrogels with grown patterns were immersed in pure water for removing residual monomers and then were observed at high temperature (50 ^{o}C) under ultraviolet light (UV) irradiation (Supplementary Figure 5).

Formation of single microstructure by micro-indentation. The measurements were carried out using an MCT-2150 mechanical tester (A&D Co., Ltd.) in a glove box for the DN hydrogel with NIPAm monomer, and using a Shimadzu Autograph AG-X 10N tensile machine in air for the DN hydrogel without monomers (Supplementary Figures 7–12). To characterize the mechanical properties of tough DN hydrogel surfaces with and without feeding monomers, the cylindrical steel indenter with a smooth cutting edge (diameter: ~568 μm) was fixed on load cell by screw, the hydrogels were cut into disc shape (diameter: ~15 mm) then glued to a rigid glass plate from Matsunami company of Japan (S2112) prior to the surface micro-indentation. The indentation velocity was 10 mm min^{-1} in the glove box and 1 mm min^{-1} in air.

Surface observation of different hydrogel surfaces and steel indenters by a three-dimensional (3D) laser microscope. Different swollen hydrogel surfaces were observed in air by a 3D laser microscope (VK-9710, Keyence Co., Ltd.). Free water on hydrogel surfaces was wiped out by clean tissues prior to observation. The magnification was 10, and the resolution was 0.5 μm for each observation. Surface roughness value R_a was calculated by selecting a

small square area (500 μm × 500 μm) on hydrogel surfaces. Side and top surfaces of steel indenters were also observed by the laser microscope, as shown in Supplementary Figure 13. Different hydrogel surfaces, microstructures, patterns, and the reswollen zones were also observed by the microscope, profile and size of microstructures were measured by a software VK Analyzer.

Contact angle measurements of the micropatterned hydrogel surfaces. The patterned hydrogel surface was washed by deionized water three times prior to the measurements. The static water contact angle of the patterned hydrogels was measured by using a Drop master 300 (Kyowa Interface Science Co., Ltd.) in air at 25 ^{o}C, water volume of 10 μL was used, free water on hydrogel surfaces was wiped off using cleaning tissues prior to each measurement, waiting time was 5 s for each measurement (Supplementary Figure 27). The dynamic contact angle measurements were also carried out using the same machine, the hydrogels were adhered to the glass plate with glue, the glass plate was placed vertically, water droplet of 10 μL was extruded vertically to the hydrogel surface by a pipette. The contact angle was the average value of three measurements.

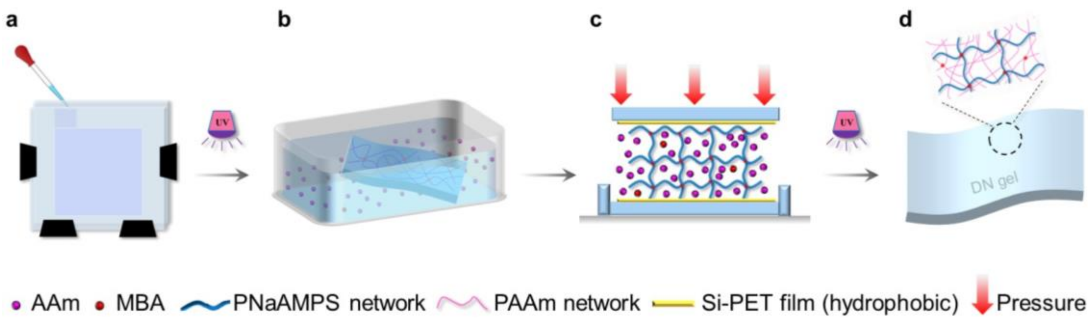

Supplementary Figure 1. Schematic illustration of fabrication of tough double-network (DN) hydrogels with the double-network structure to the top-most surface layer. **a**. The first network hydrogel was polymerized in a glass mould under UV light. **b**. The prepared first network hydrogel was immersed in the AAm monomer aqueous solution. **c**. A hydrophobic silicone-coated PET (Si-PET) film was covered on the glass mould surface by pressure (~0.8 kPa) during the second network synthesis to completely remove surface solution (residual AAm monomer aqueous solution), which also prevented the formation of the electric double layer at PNaAMPS-glass interface. **d**. By this method, the PNaAMPS hydrogel is in contact with the hydrophobic surface of the Si-PET film, which ensures the formation of DN structure up to the top-most surface.

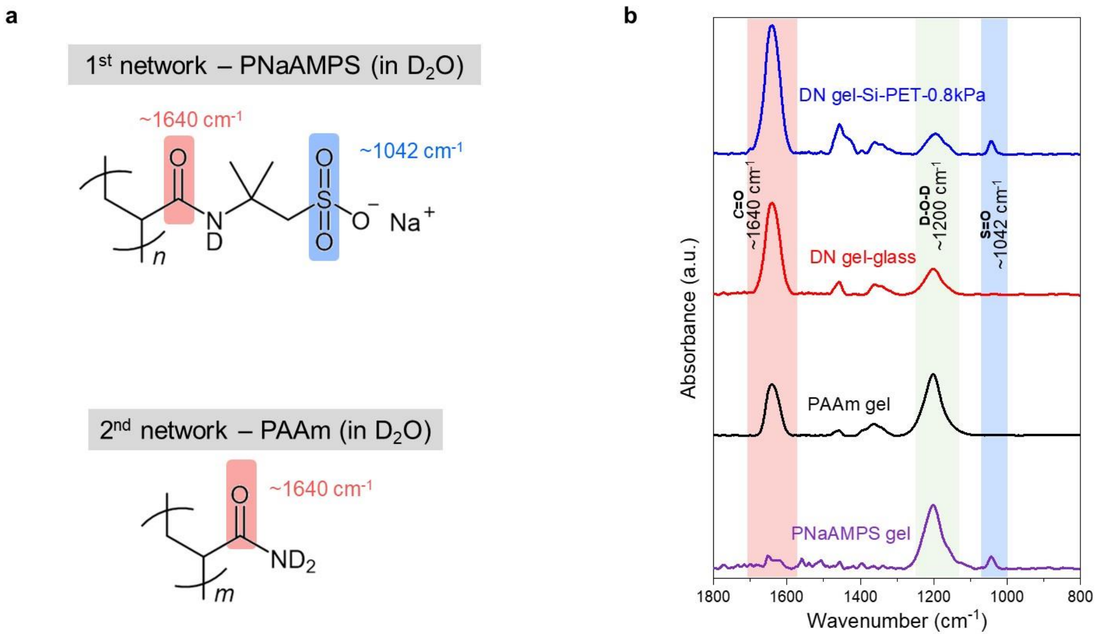

Supplementary Figure 2. a. Chemical structures of polymer networks in the double network hydrogels and the functional groups used for surface characterization. **b**. Attenuated total reflectance Fourier-transform infrared spectroscopy of single-network and DN hydrogels. DN hydrogel of its second network prepared on the Si-PET film covered glass mould (coded DN gel-Si-PET-0.8kPa) shows the obvious PNaAMPS signal at ~1042 cm^{-1}, while DN hydrogel of its second network prepared on glass mould (coded DN gel-glass) does not. These results indicate that the DN structure is formed even at the top-most surface in the former DN hydrogel while the surface of the latter DN hydrogel is covered by PAAm.

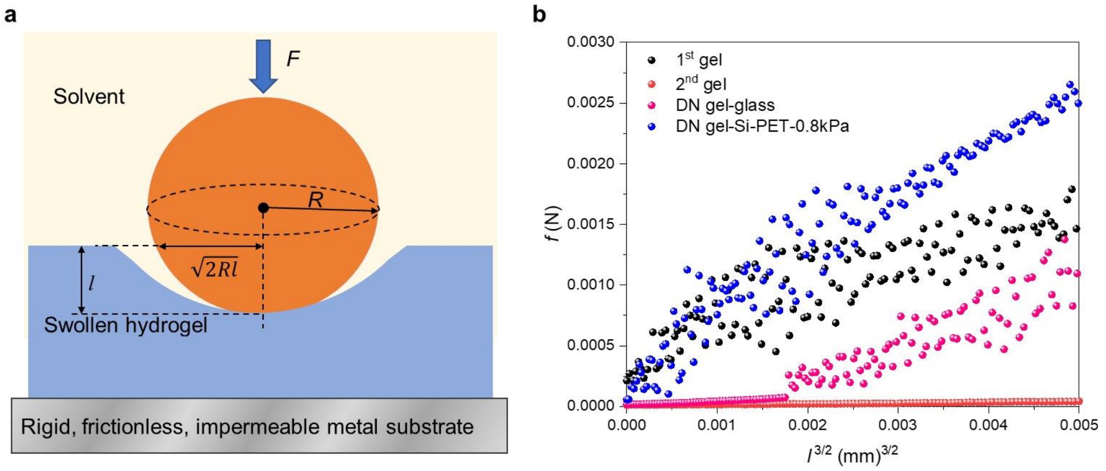

Supplementary Figure 3. a. Schematic illustration for hydrogel surface indentation measurements under water using a sphere metal indenter. Hydrogel thickness was 3.5–5 mm, which was far larger than the indentation depth (~0.029 mm) in the experiments. **b.** Indentation curves of f-$l^{3/2}$ for different indentation measurements, the slopes of curves are proportional to the surface elastic moduli of different hydrogels. DN hydrogels with its second network prepared on Si-PET film covered glass mould show the almost same modulus as the single first network, while DN hydrogels with its second network prepared on glass mould show a lower modulus than the single first network. These results again indicate that the double-network structure is formed even at the top-most surface in the former DN hydrogel while the surface layer of the latter DN hydrogel is covered by the soft second network. These results are consistent with the IR results shown in Supplementary Figure 2.

Supplementary Table 1. Surface elastic modulus of the different hydrogels (1st gel is PNaAMPS SN gel), 2nd gel is PAAm SN gel, DN gel-glass and DN gel-Si-PET-0.8kPa are DN hydrogels with different surfaces.

Hydrogels	1st gel	2nd gel	DN gel-glass	DN gel-Si-PET-0.8kPa
Surface elastic modulus (kPa)	661.2 ± 88.9	6.6 ± 0.1	14.2 ± 0.3	683.2 ± 52.9

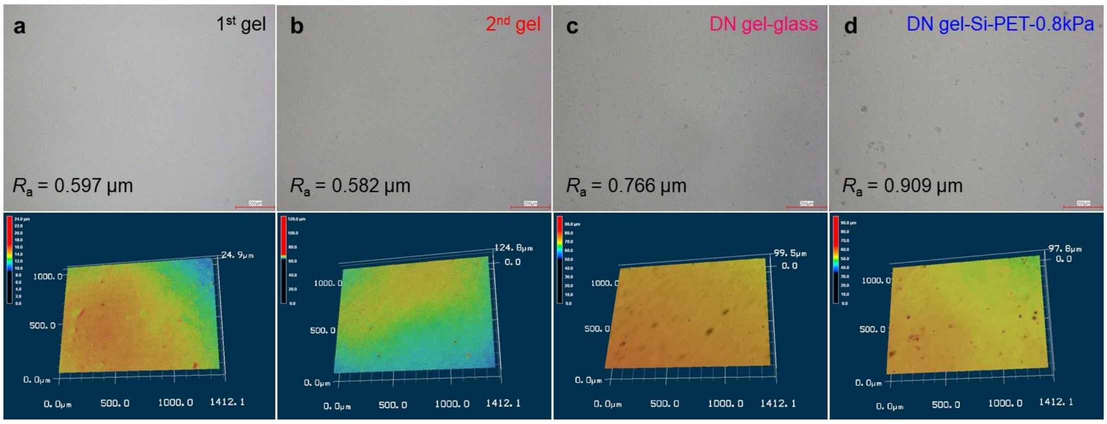

Supplementary Figure 4. Surface topography (roughness) of different hydrogels observed by 3D laser microscope in air. Here, R_a was calculated as the roughness average of hydrogel surfaces measured microscopic peaks and valleys. **a**. 1st gel (PNaAMPS SN gel). **b**. 2nd gel (PAAm SN gel). **c**. DN gel-glass (DN hydrogel prepared between glasses). **d**. DN gel-Si-PET-0.8kPa (the second network of DN hydrogel was prepared between silicon-coated PET film under the pressure of ~0.8 kPa). Scale bars, 200 µm.

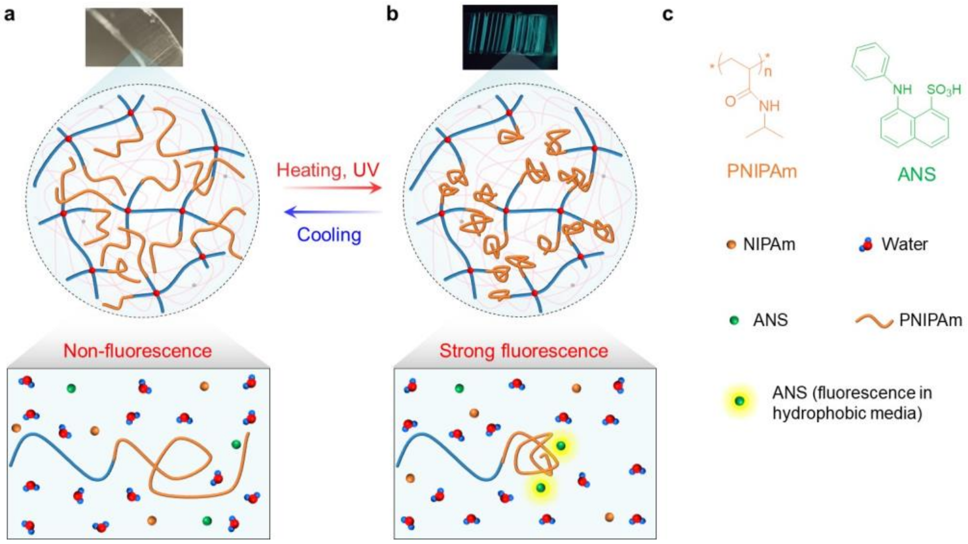

Supplementary Figure 5. Molecular mechanism of fluorescence emission from the patterned region on DN hydrogel surfaces. **a**. The patterned region of DN hydrogel hardly exhibits fluorescence at 25 °C. **b**. The PNIPAm patterned region was visualized when observed at high temperature (50 °C) that exceeds the lower critical solution temperature (32 °C) of PNIPAm, because the fluorescent molecule ANS exhibits strong fluorescence only in the hydrophobic environment. **c**. Molecular structures of PNIPAm and ANS[7].

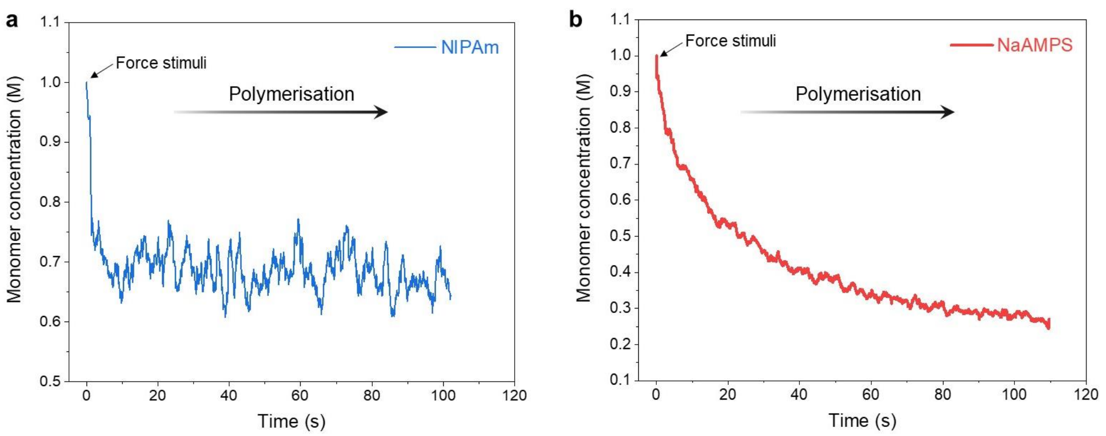

Supplementary Figure 6. Kinetics of the force-triggered radical polymerisation. The time-resolved near-infrared spectroscopy revealed that the force-triggered radical polymerisation almost completed within tens of seconds. **a**. Time-resolved concentration of NIPAm versus time. **b.** Time-resolved concentration of NaAMPS versus time. Here, the curves of time-resolved near-infrared spectroscopy were the average value of at least two replicates.

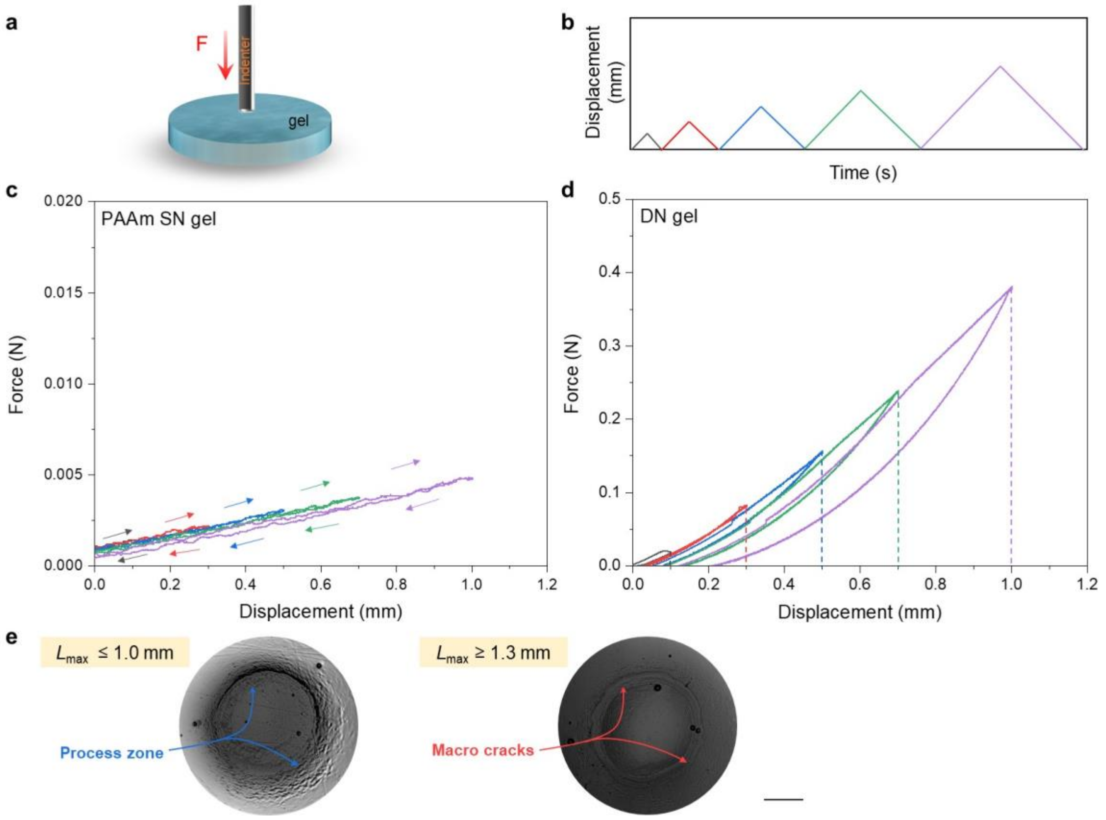

Supplementary Figure 7. Cyclic indentation on hydrogels using a cylindrical steel indenter (diameter *d* = 568 μm) in air. **a**. Schematic illustration for the cyclic indentation. **b**. Applied displacement changes over time for the cyclic indentation. **c**. Force-displacement curves show that PAAm SN gel surface is nearly elastic, PAAm gel thickness was ~5 mm. **d**. Force-displacement curves show that DN hydrogel surface is a tough surface with pronounced hysteresis, hydrogel thickness was ~3.8 mm. **e**. Microscope images of the process zones on DN hydrogel surfaces. When displacement L_{max} was increased beyond 1.0 mm, macrocracks appeared on DN hydrogel surfaces, which was marked by the arrow. Accordingly, the maximum displacement of intender without macroscopic damage was 1.0 mm. The displacement velocity was 1 mm min^{-1}. Error bars represent standard deviation from three replicates. Scale bar, 200 μm.

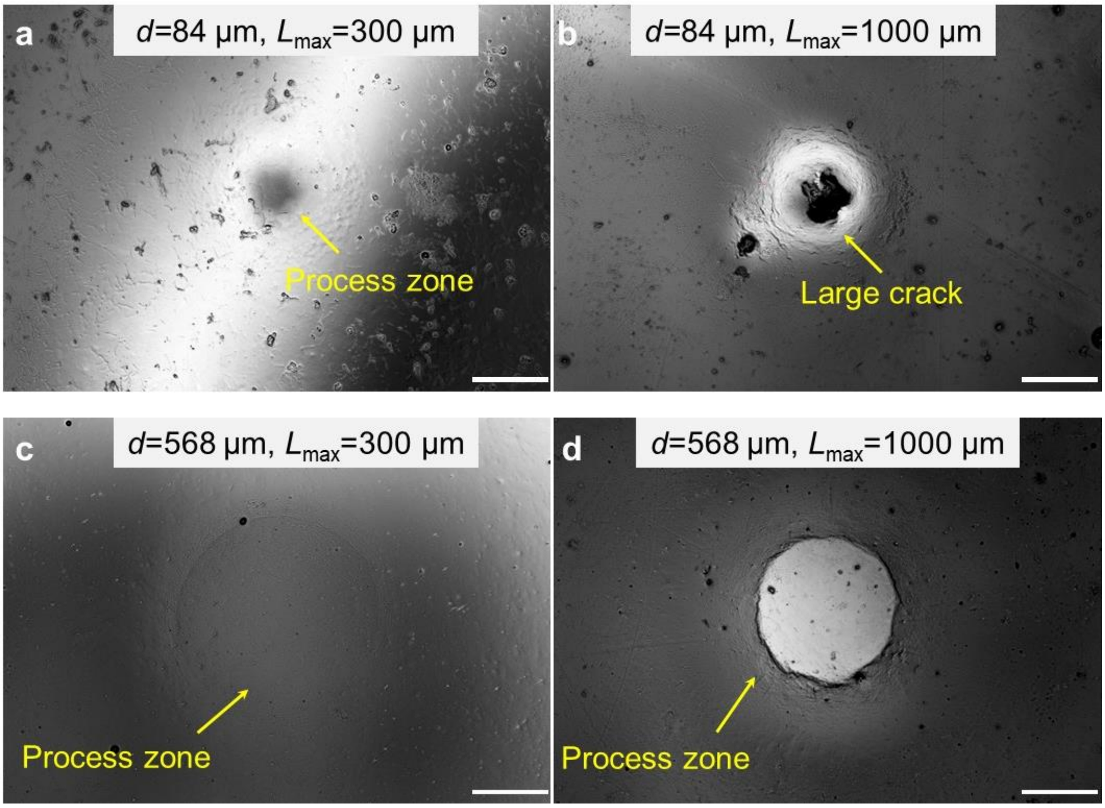

Supplementary Figure 8. Microscope images of the process zones on DN hydrogel surfaces. **a**. A small process zone appeared on DN hydrogel surface when indenter diameter of 84 μm and indentation depth of 300 μm were used for surface indentation. **b**. When indentation depth L_{max} was increased to 1000 μm, large crack (or damage hole) appeared on DN hydrogel surface, which was marked by the yellow arrow. Scale bar for **a, b** is 100 μm. **c**. A large process zone appeared when big indenter diameter of 568 μm and indentation depth of 300 μm were used for indentation. **d**. An obvious process zone appeared when indentation depth was increased to 1000 μm, without showing microcrack. Scale bar for **c, d** is 200 μm.

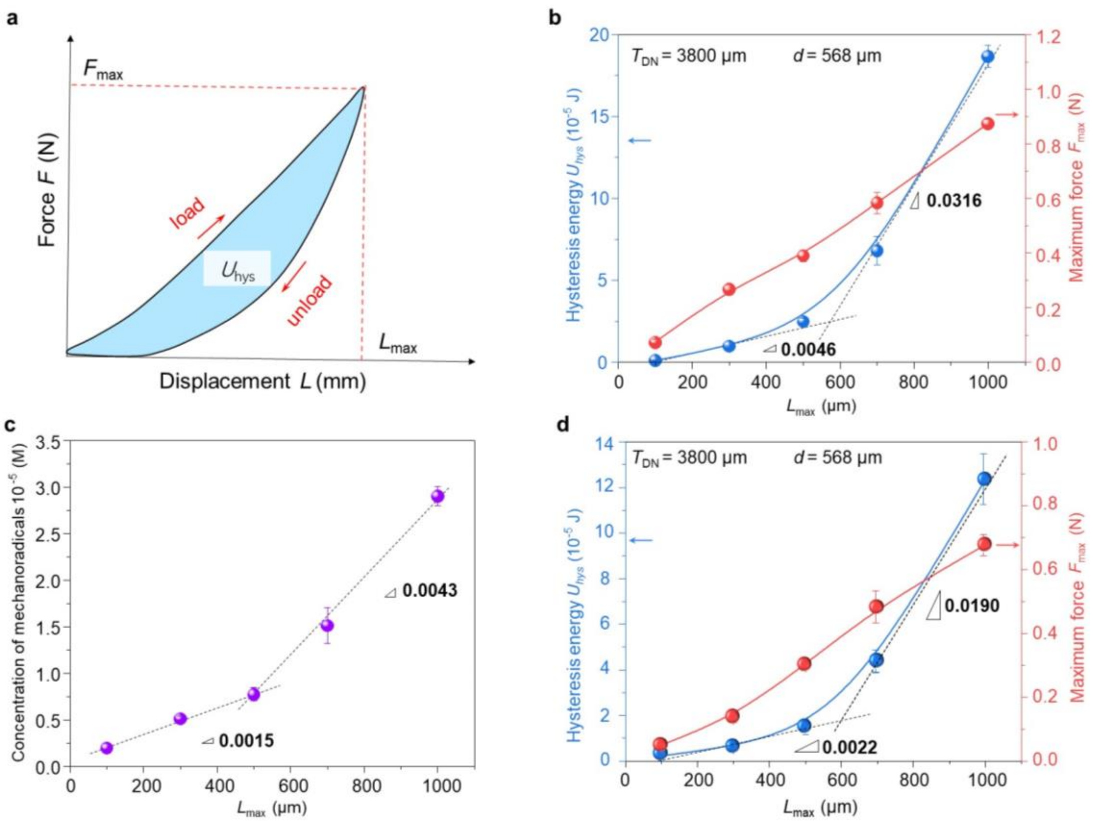

Supplementary Figure 9. a. Illustration of quantification of the mechanical energy U_{hys} dissipated by cyclic indentation on tough DN hydrogel surfaces using a cylindrical steel indenter. The light blue area beneath the force-displacement curve gives the work done U_{hys} by mechanical force to the DN hydrogel surfaces. The U_{hys} was estimated from $U_{\mathrm{hys}} = \int_0^{L_{max}} (F_{\mathrm{load}} - F_{\mathrm{unload}})\mathrm{d}L$. **b**. The hysteresis energy U_{hys} and maximum force F_{max} measured at various indentation displacements L_{max} by mechanical indentation in the air without monomer supply (a cylindrical steel indenter diameter was d = 568 μm, the velocity of indentation was 1 mm min^{-1}, DN hydrogel thickness was ~3.8 mm). **c**. The hysteresis energy changed with indentation displacement L_{max}. The dissipated energy per mole of strands of the first network was estimated based on the analysis by Lake and Thomas[8]. Here, the mechanical energy required to break one C-C covalent bond, ΔU_{act}, was considered to be 25% of the C-C bond dissociation energy E_b (~350 kJ/mol). The number of C-C bonds in the bridging strands of the first network, N, was estimated by using the affine network theory[9]. Here, N for the first network in the current study (synthesized from 1.0 M NaAMPS and 40 mM MBA) was estimated to be ~580 according to our previous work[10,11]. The process zone volume was assumed as $\pi(d/2)^2 L_{max}$. For example, when L_{max} was 1000 μm, the volume of process zone was $\pi(568\ \mu m/2)^2 \times 1000\ \mu m = 2.53\times10^{-7}\ dm^3$, and the hysteresis energy U_{hys} was 1.87×10^{-4} J. The mechanical energy U required to break a polymer strand with N monomers was estimated as $U = N\Delta U_{act} \approx 580 \times (350\ kJ\ mol^{-1} \times 0.25) = 5.1\times10^{7}\ J\ mol^{-1}$. The number of moles of mechanoradical was $2(U_{hys}/U) = 7.4\times10^{-12}$ mol, and the mechanoradical concentration was roughly estimated as $(7.4\times10^{-12}\ mol)/(2.5\times10^{-7}\ dm^3) = 2.9\times10^{-5}\ mol\ dm^{-3}$ or 2.9×10^{-5} M. For comparison, the $U_{hys} \sim L_{max}$ results for DN hydrogel fed with monomer NIPAm (1.0 M) were also shown in **d**. Since with

the presence of monomer, the energy dissipation might contain a complex mechanism, we did not estimate the mechanoradical concentration from the energy dissipation in this case. Error bars in **b-d** represent the standard deviation from three replicates. Some error bars in **b-d** are hidden by the symbols.

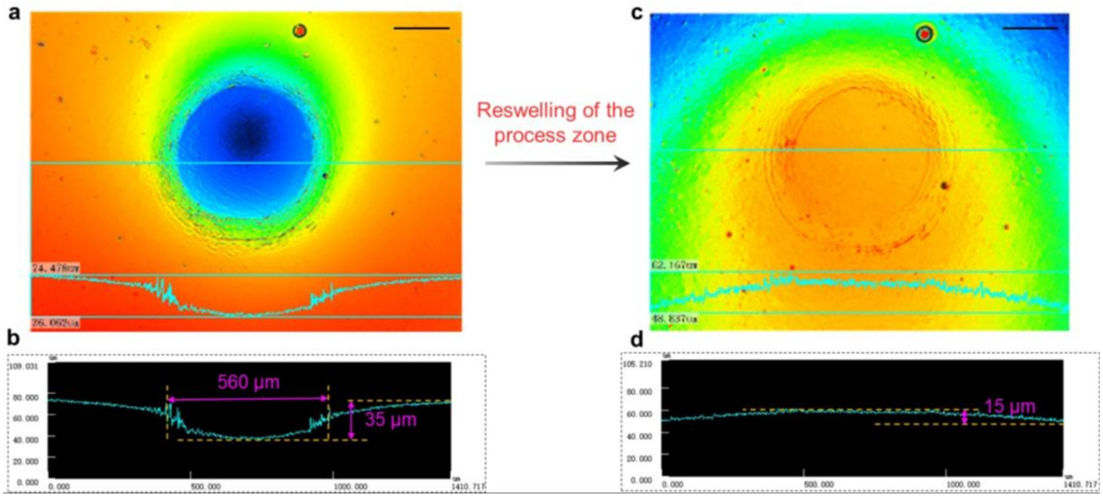

Supplementary Figure 10. Reswelling of the process zone (or stressed region) on DN hydrogel surfaces. Immersion time in water for the reswelling was several seconds. **a**. Image of the process zone on DN hydrogel surface. **b**. Profile and size of the process zone. **c**. Image of the reswollen zone on DN hydrogel surface. **d**. Profile and height of the reswollen zone. Scale bars, 200 μm. A cylindrical steel indenter of diameter d = 568 μm and displacement L_{max} = 1000 μm, DN hydrogel thickness was around ~3.8 mm.

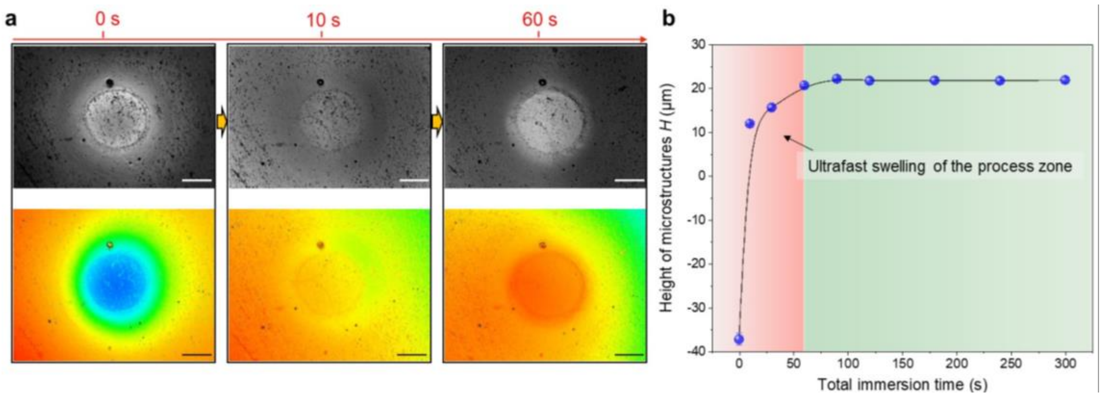

Supplementary Figure 11. Observation of the fast swelling on DN hydrogel surface, the process zone was created by regioselective indentation under deionized water without monomer (cylindrical steel indenter diameter was ~568 μm, displacement was ~1000 μm). **a**. The process zone morphology changed over the immersion time. The area of process zone was around ~0.1 mm^2. **b**. Height of reswollen microstructure changed over the immersion time (time scale of equilibrium swelling was approximately 60 s). The height H of a DN hydrogel surface for different total immersion times t were characterized as follows. First, an indented DN hydrogel surface was observed by the microscope in air (t = 0 s). The hydrogel was then immersed in water for 10 s, picked up from water and wiped by clean tissues, then observed by the microscope in air (t = 10 s). Afterwards, the hydrogel was immersed in water for 20 s followed by the microscope observation in the same manner (total immersion time t = 10 + 20 = 30 s). Such protocol was repeated several times for this DN hydrogel surface, resulting in H as a function of t up to 300 s. The result indicates that the time scale of equilibrium swelling was about 60 s. The light red shading in **b** indicates the fast swelling of the damage zone, the light green shading indicates the equilibrium process. Error bar represents the standard deviation for three replicates. Some error bars in **b** are hidden by the symbols. Data in **b** are presented as mean values +/- SD. Scale bars, 200 μm.

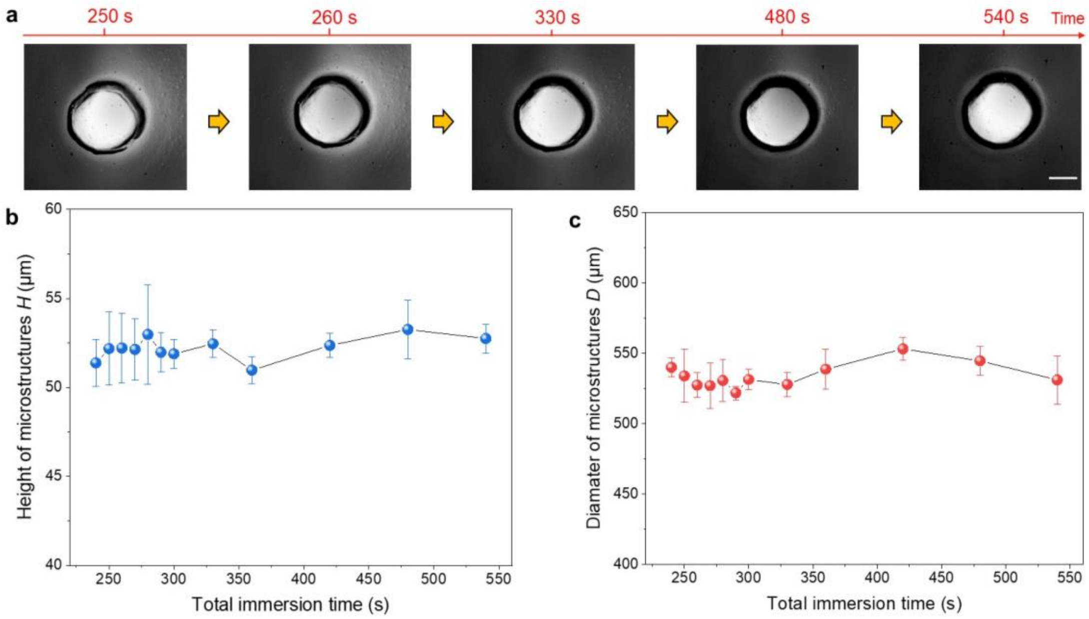

Supplementary Figure 12. Evolution of microstructures on DN hydrogel surface in monomer aqueous solution. The microstructures were created by regioselective indentation under NIPAm aqueous solution (cylindrical steel indenter diameter was ~568 μm, displacement was ~1000 μm, indentation speed was 10 mm min^{-1}, the concentration of NIPAm was 1.0 M). **a**. Microstructure morphology changed over immersion time. Scale bar, 200 μm. **b**. Height of microstructures changed over immersion time. **c**. Diameter of microstructures changed over immersion time. The height H and diameter D of a microstructure on DN hydrogel surface for different total immersion times t were characterized as follows. First, a DN hydrogel was indented under the monomer solution in the glove box. The indented hydrogel in the solution was immediately taken out from the glove box, then the hydrogel was observed by the microscope in air after wiping the solution on the hydrogel surface by clean tissues. The time between the end of the indentation and the first microscope observation was 4 min (because of physical distance from the glove box to the microscope), during which the hydrogel was kept in the solution. Hence, we assumed the immersion time t of 240 s for the first observation. After the first observation, the hydrogel was immersed again in the solution for 10 s, picked up from solution and wiped by clean tissues, then observed by the microscope in air (total immersion time t = 240 +10 = 250 s). Such step of immersion and observation was repeated several times for this DN hydrogel surface, resulting in H and D as a function of t up to 540 s. The results show that the micropatterning was finished before the first observation at t = 240 s. Error bars represent the standard deviation from three replicates. Data in **b** and **c** are presented as mean values +/- SD.

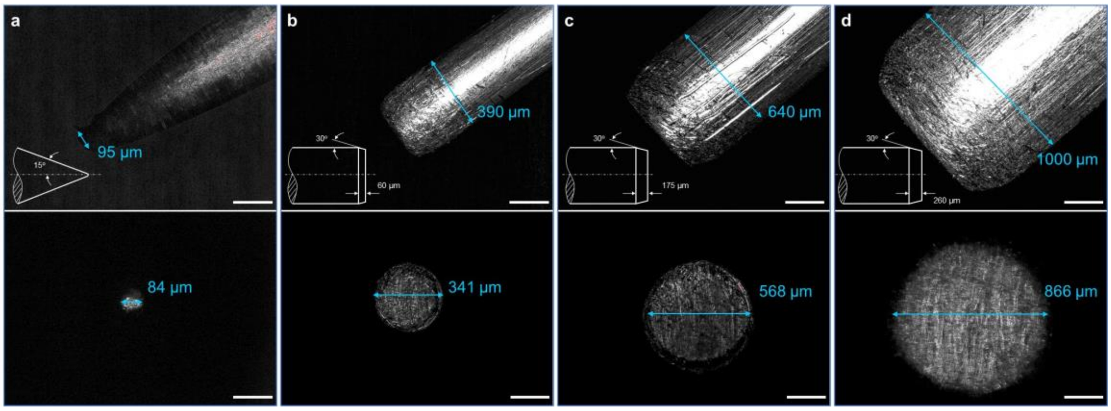

Supplementary Figure 13. a-d. Microscope images of the cylindrical steel indenters with different diameters. The side view of the indenters (upper row). Insets, schematic diagram of engineering drawing for the cylindrical steel indenters. The bottom view of indenters (lower row). Scale bars, 200 μm.

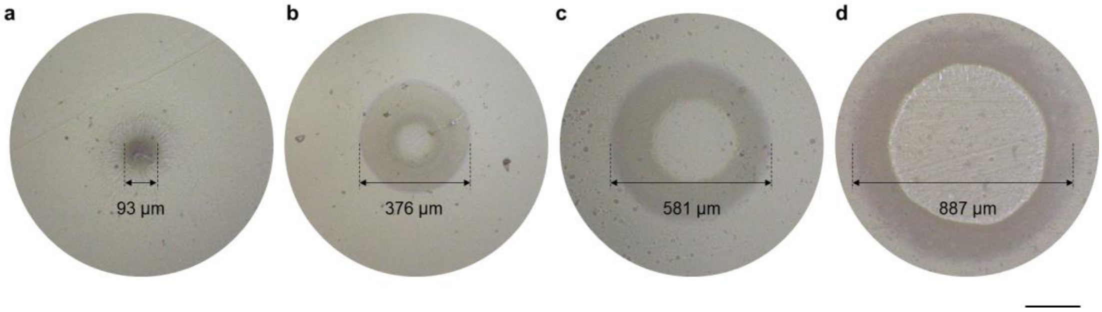

Supplementary Figure 14. a-d. Microscope images of the microstructures on DN hydrogel surfaces, which were indented with different cylindrical steel diameters (see Supplementary Figure 13). The scale and shape of microstructures depends on the indenters. The experiments were carried out in NIPAm aqueous solution (NIPAm: 1.0 M) under argon atmosphere. The indenter diameters were 84, 341, 568, and 866 μm for a, b, c, and d, respectively. The indentation depth L_{max} was 1000 μm. Scale bar, 200 μm.

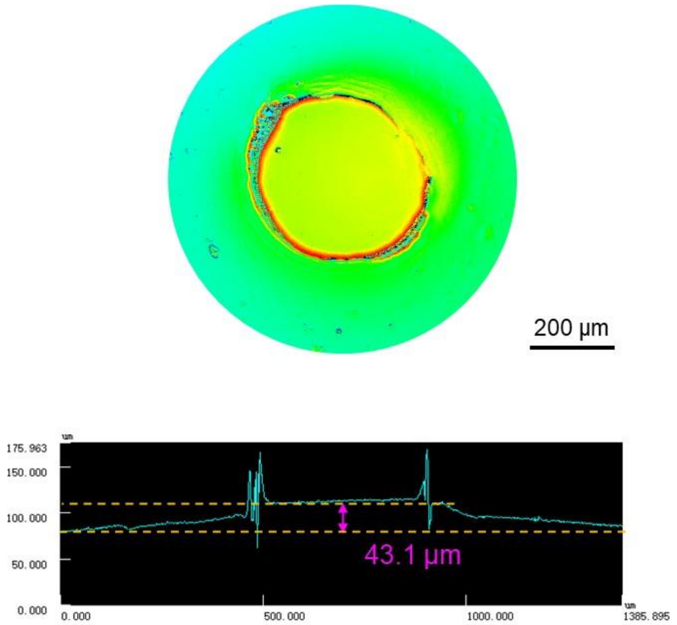

Supplementary Figure 15. Microscope image of the grown microstructure on the double-network hydrogel surface (coded DN gel-glass, with PAAm layer), the topological height is 43.1 µm. Here, monomer NaAMPS was 1.0 M, a cylindrical steel indenter of diameter *d* = 568 µm and displacement L_{max} = 1000 µm were used for the force-triggered polymerization in hydrogels, DN hydrogel thickness was around ~3.8 mm.

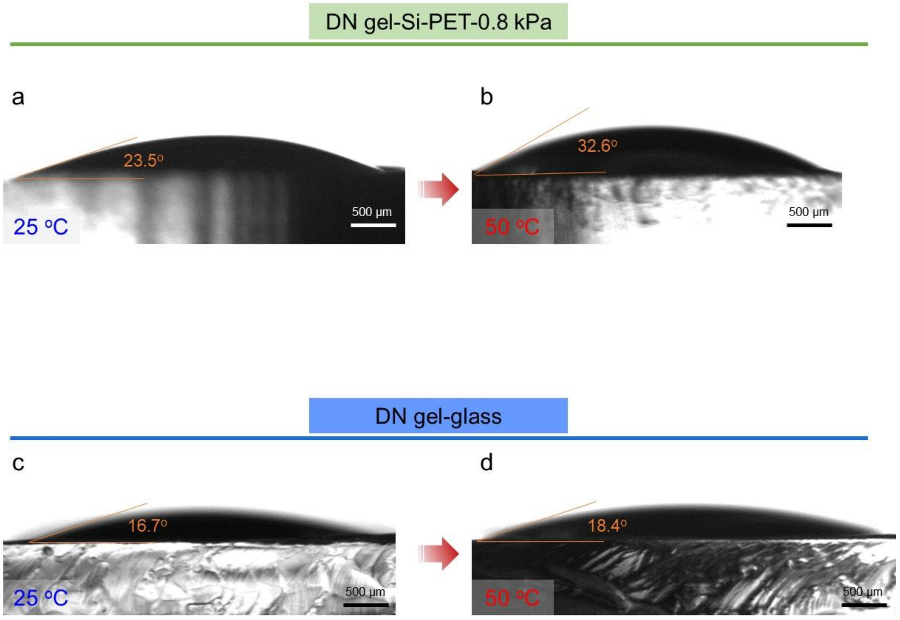

Supplementary Figure 16. a, **b**. Optical images of a water droplet (10 µL) on the modified surface of the DN gel-Si-PET-0.8kPa. The contact angle of the water droplet on the surface is significantly increased at higher temperature. **c**, **d**. Optical images of a water droplet (10 µL) on the modified surface of the DN gel-glass. The contact angles of the water droplet on the surface are almost the same at different temperatures. Here, the cylindrical steel indenter with bigger diameter of 15 mm was used, the indentation depth was 1000 µm.

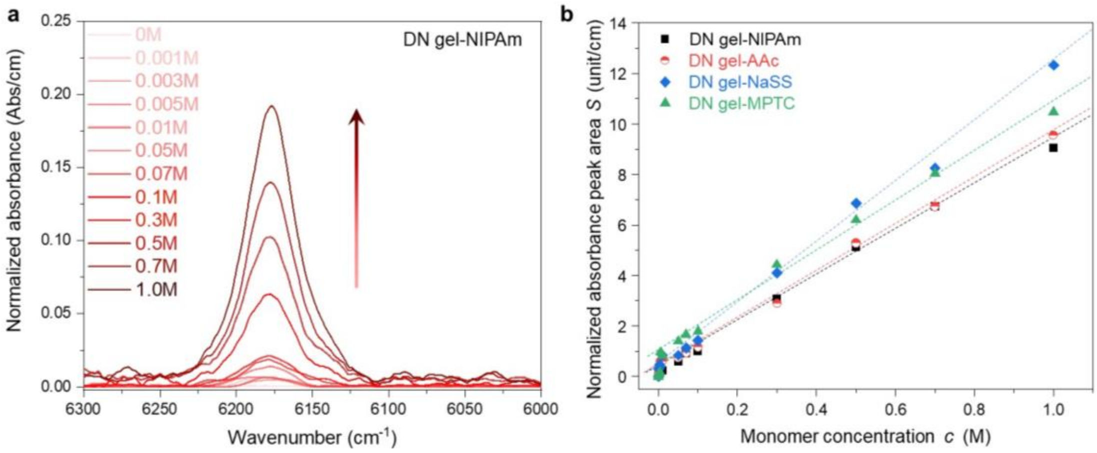

Supplementary Figure 17. a. Transmission near-infrared spectrum of DN hydrogels fed with NIPAm (coded DN gel-NIPAm) of different concentrations. DN hydrogels were equilibrated in large amount of NIPAm solution of varied concentration (0–1.0 M). The characteristic absorption peak at wavenumber 6200–6100 cm^{-1} corresponds to C–H overtone stretching at the C=C bond of monomers. **b**. The relevant calibration curves for different monomers. The results indicate that there is a linear correlation between the normalized absorbance peak area *S* and the monomer concentration *c*.

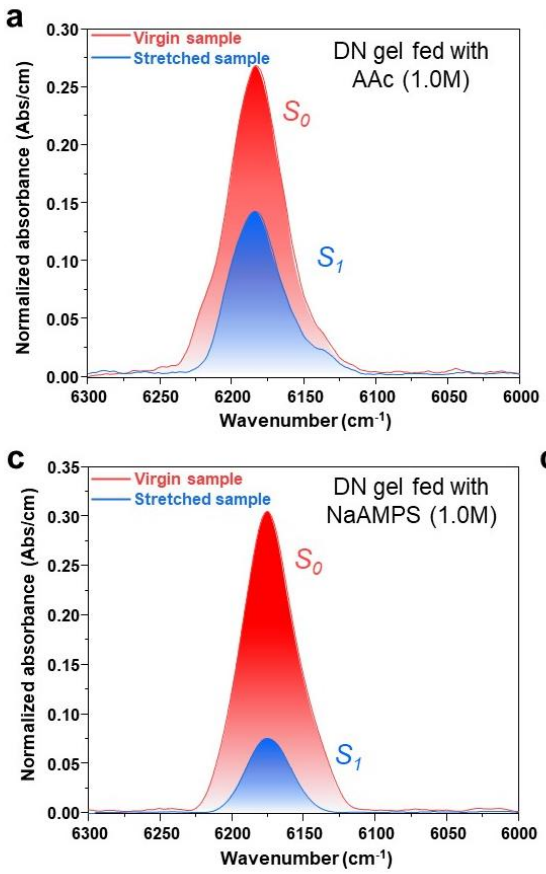

Supplementary Figure 18. Transmission near-infrared spectra of DN hydrogel fed with different monomers before stretching (peak area, S_0) and after stretching (peak area, S_1), measurement waiting time was 1 min, the stretched strain ε was 3 (over yielding). **a**. AAc, **b**. NaSS, **c**. NaAMPS, **d**. MPTC. Initial monomer concentration was 1.0 M, and crosslinker (MBA) was not used in this experiment.

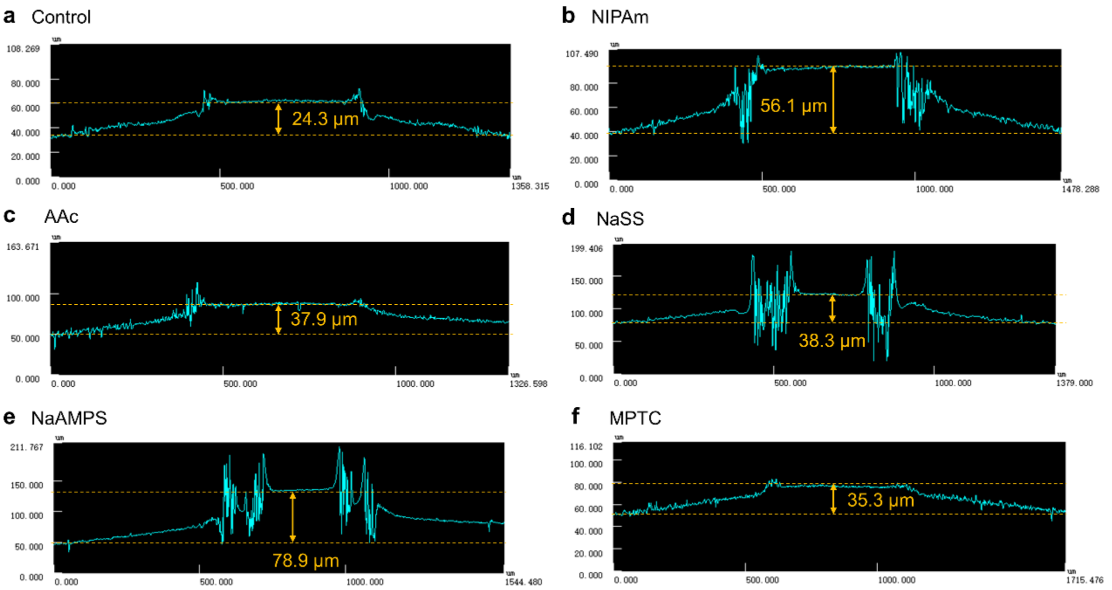

Supplementary Figure 19. a-f. The typical profiles of the microstructures on DN hydrogel surfaces using different functional monomers, the control sample was prepared without monomer and crosslinker supply. Monomer concentration was 1.0 M without crosslinker for all cases except for the control.

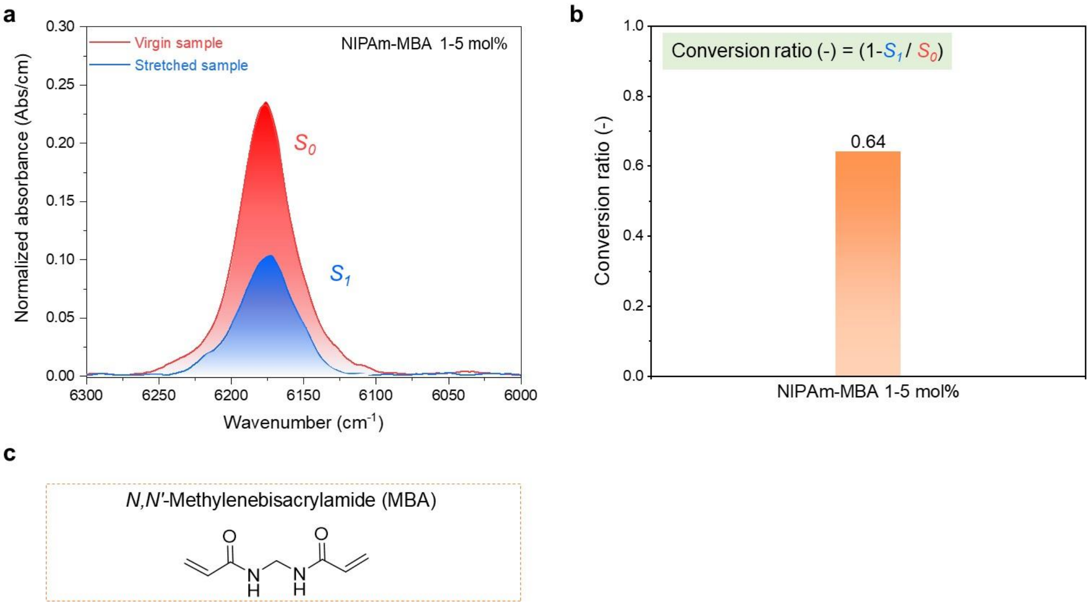

Supplementary Figure 20. a. Transmission near-infrared spectra of DN hydrogel fed with NIPAm monomer and crosslinker MBA before stretching (peak area, S_0) and after stretching (peak area, S_1), measurement waiting time was 1 min. NIPAm monomer concentration was 1.0 M, the molar ratio of crosslinker MBA respect to NIPAm monomer was 5 mol%. **b**. The conversion ratio for DN hydrogels fed with NIPAm and MBA. Here, conversion ratio was calculated based on the inset equation. **c**. Molecular structure of *N,N'*-methylenebisacrylamide (MBA).

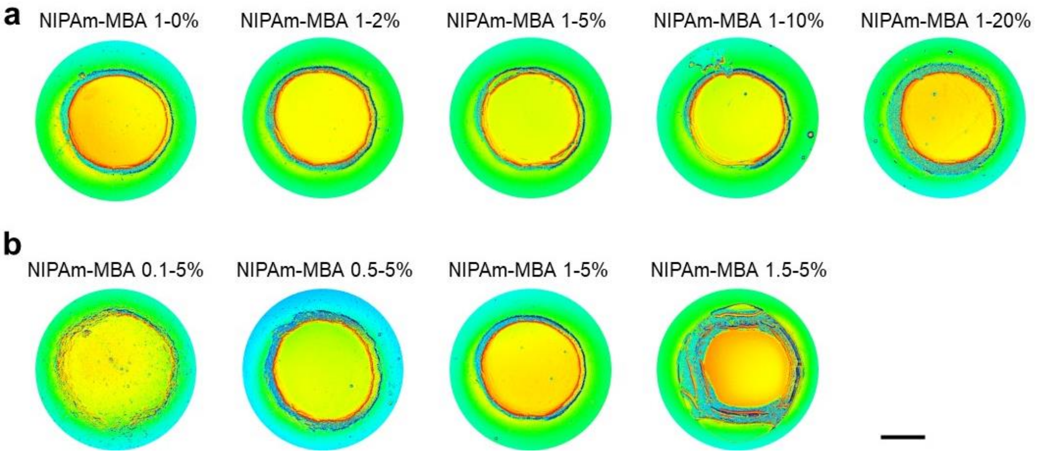

Supplementary Figure 21. a. Three-dimensional laser microscopy observation of the microstructures using different crosslinker concentrations (sample NIPAm-MBA 1-*X*% indicates that the monomer NIPAm concentration was 1.0 M and crosslinker MBA concentration was *X* mol% in relative to the NIPAm). **b**. Three-dimensional laser microscopy observation of the microstructures using different monomer concentrations. The experiments were performed at 25 °C. Scale bar, 200 μm.

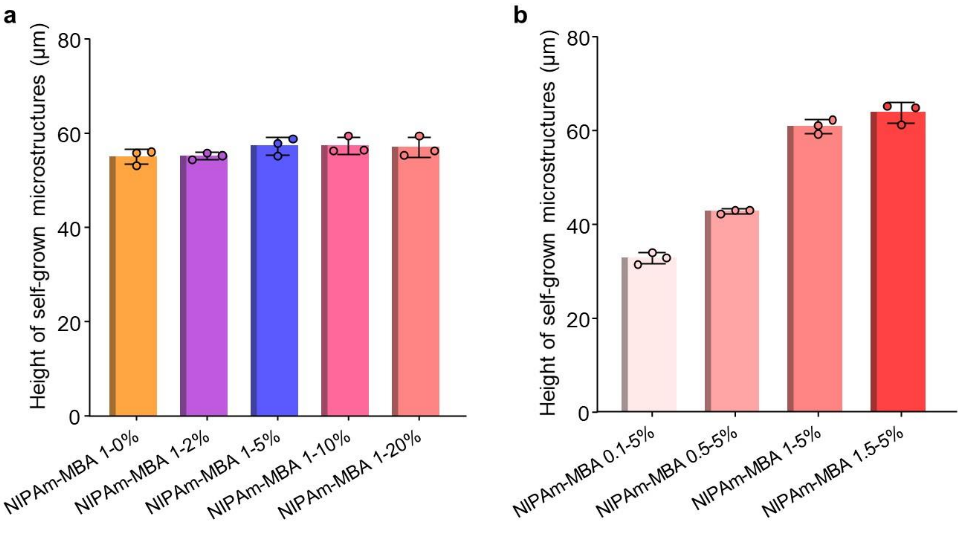

Supplementary Figure 22. a. The height of microstructures using different crosslinker ratios (Here, the percentage was in relative to monomer molar concentration of 1.0 M). **b**. The height of microstructures using different monomer concentrations at a fixed crosslinker density of 5 % in relative to monomer molar concentration. The experiments were performed at 25 °C. Error bar represents the standard deviation for three replicates. The circular dots of the histogram in **a** and **b** represent the measured data points of three. Data in **a** and **b** are presented as mean values +/- SD.

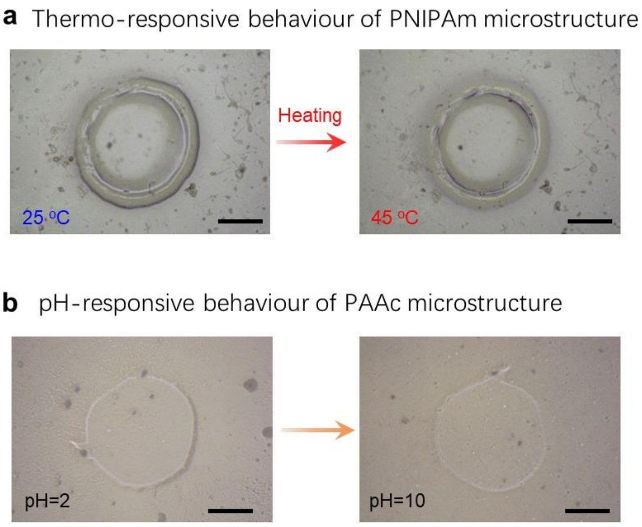

Supplementary Figure 23. a. Microscope images of PNIPAm microstructure at different temperatures (with water in air), the change in feature height of microstructure was controlled by thermo-induced swelling and deswelling, NIPAm of 1.0 M without crosslinker was used. Scale bars, 200 μm. **b**. Microscope images of PAAc microstructure immersed in solutions with different pH (the buffer solution (pH=2) was 10^{-2} M HCl aqueous solution, the buffer solution (pH=10) was 10^{-4} M NaOH aqueous solution, the temperature was 25 °C). the change in feature height of microstructure was controlled by pH-induced swelling and deswelling, AAc of 1.0 M without crosslinker was used. Scale bars, 200 μm.

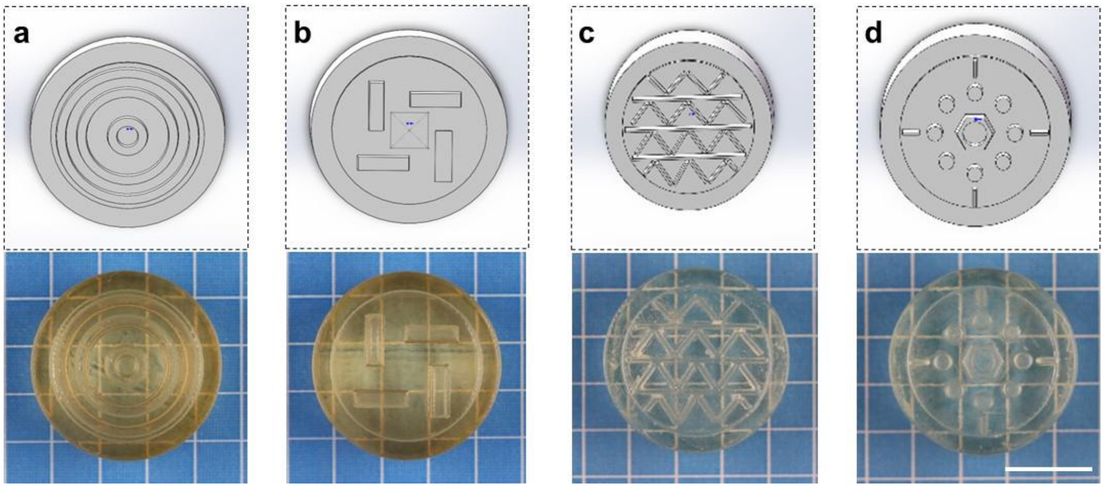

Supplementary Figure 24. a-d. Illustration of customized complex patterns for 3D printing (upper row) and photos of 3D printed resin stamps (lower row). The topographic height of all the 3D printed patterns was ~2 mm. Scale bar, 10 mm.

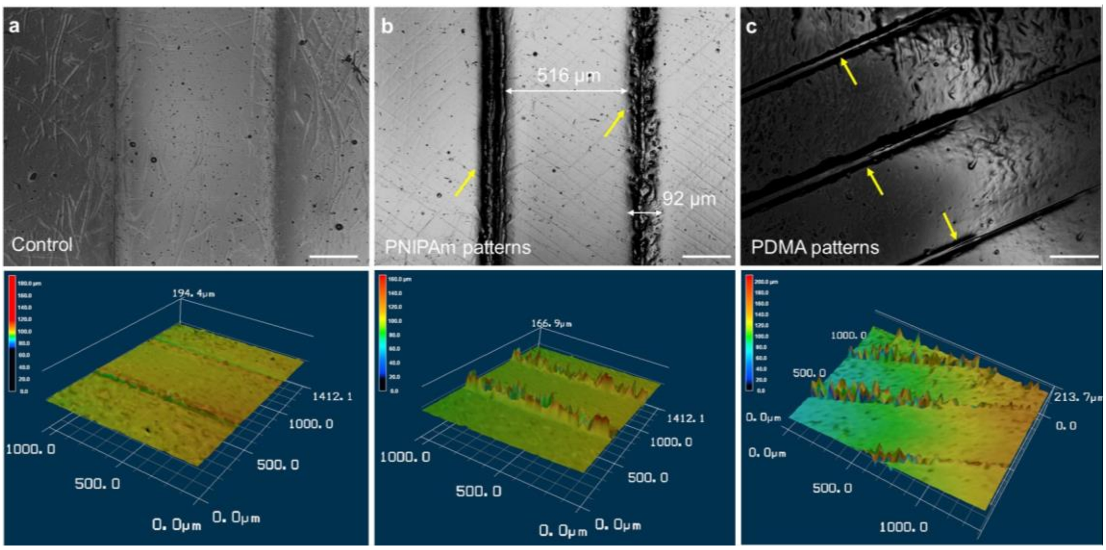

Supplementary Figure 25. Optical microscope (upper row) and three-dimensional topography images (lower row) of DN hydrogels with parallel micropatterns created by force-triggered microstructure growth. **a**. Control sample (damage the hydrogel surface under deionized water, without monomer supply). **b**. PNIPAm patterns on DN hydrogel surface (monomer NIPAm of 1.0 M was used). **c**. PDMA patterns on DN hydrogel surface (monomer DMA of 1.0 M was used). The hydrogel surfaces were washed three times by deionized water prior to the microscope observation. The yellow arrows in **b** and **c** show the parallel rough patterns on DN hydrogel surfaces. Scale bars, 200 μm. The observations were performed at 25 ^{o}C.

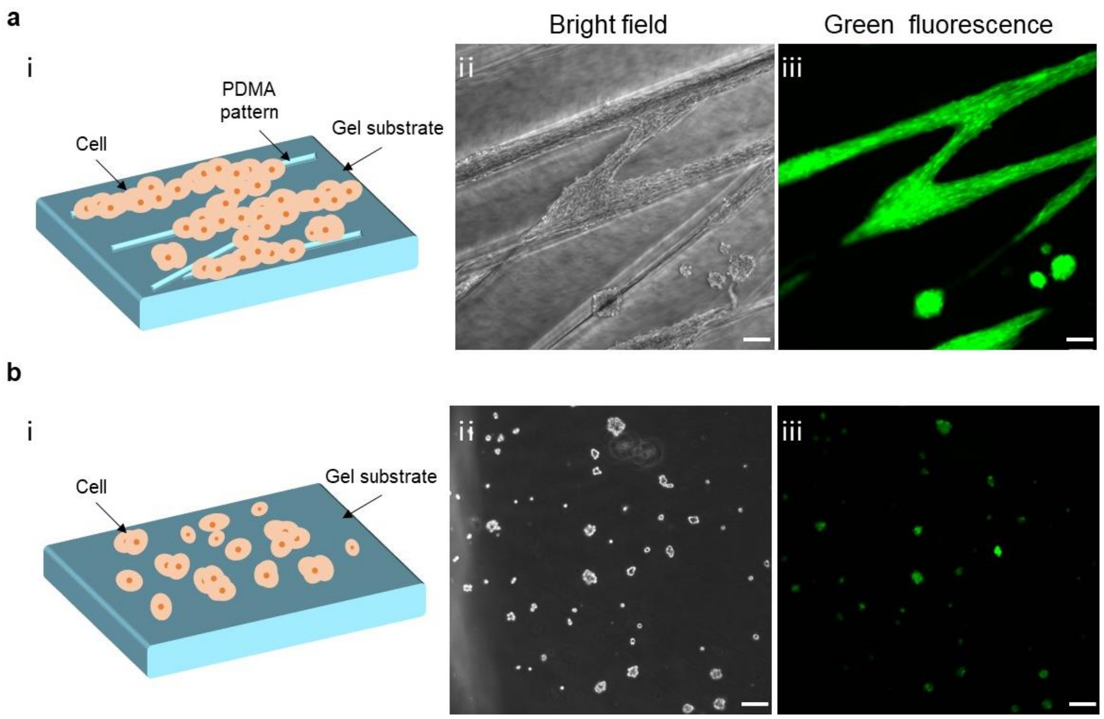

Supplementary Figure 26. a. Microscope images show that the micropatterns guide cell population orientation when cultured on the PDMA patterned DN hydrogel surfaces. **b**. Control experiment to show that the cells grow randomly on the smooth DN hydrogel surfaces. Myoblast cells labeled with a green fluorescence protein (GFP) were cultured on the hydrogel surfaces at 37 °C for 5 days. Scale bars, 100 μm.

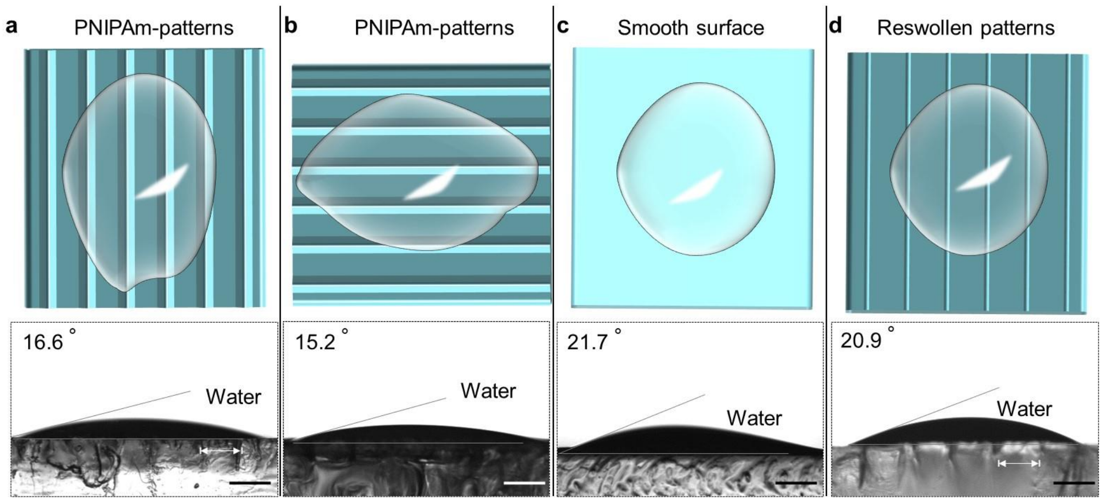

Supplementary Figure 27. a-d. Illustrations of water droplet (upper arow) and optical images of static water contact angles (lower row) on different hydrogel surfaces placed horizontally. The white arrows in optical images in **a** and **d** indicate the distance (~500 μm) between the two stripes of patterns. The water droplet was 10 μL, room temperature 25 °C. Scale bars, 500 μm.

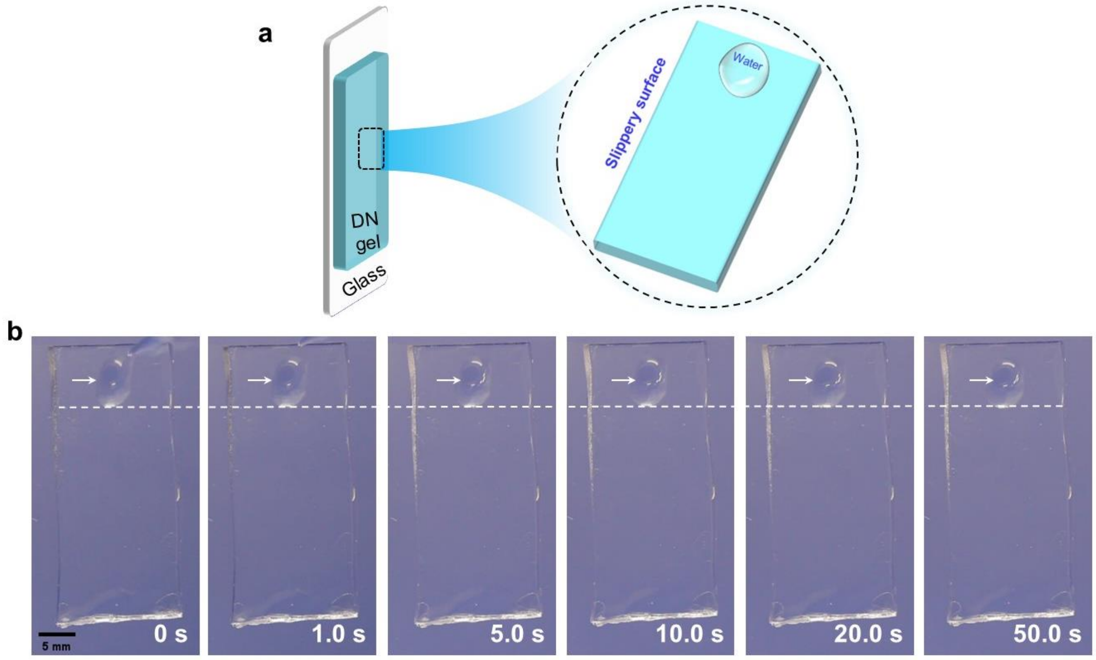

Supplementary Figure 28. a. Schematic presentation of a water droplet on smooth hydrogel surface. **b**. Images of a water droplet on smooth DN hydrogel surface. Here, the water droplet was 10 μL.

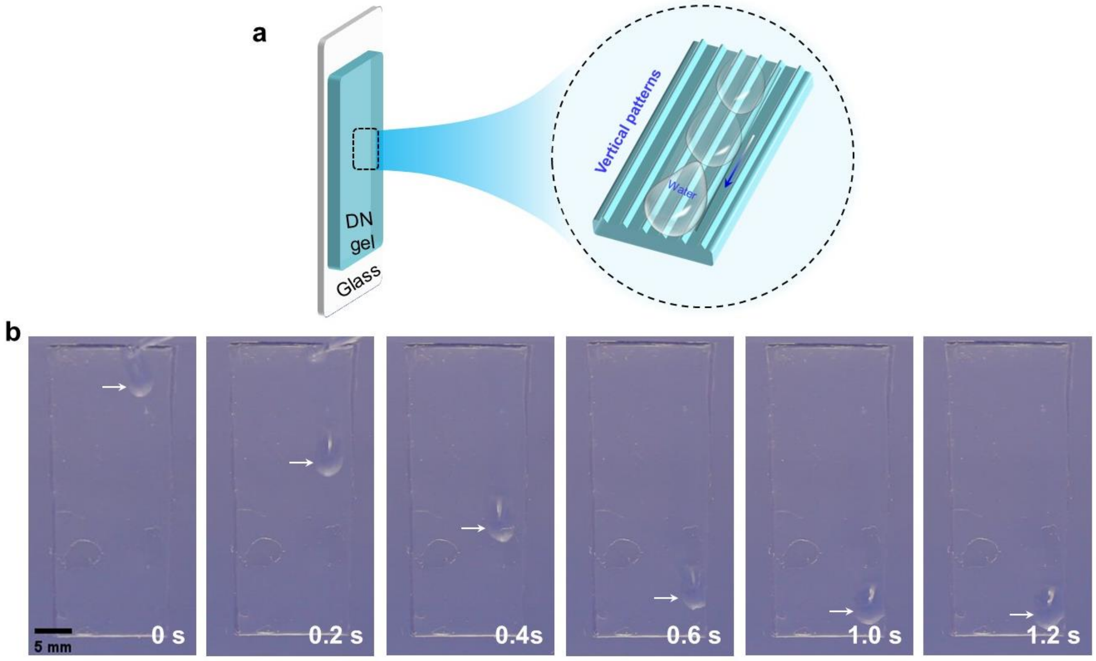

Supplementary Figure 29. a. Schematic presentation of a water droplet on DN hydrogel surface with vertical PNIPAm patterns. **b**. Water droplet fast transport on the patterned hydrogel surface. Here, the water droplet was 10 μL.

Supplementary References

1. Frauenlob, M., King, D. R., Guo, H. L., Ishihara, S., Tsuda, M., Kurokawa, T., Haga, H., Tanaka, S. & Gong, J. P. Modulation and characterization of the double network hydrogel surface-bulk transition. *Macromolecules* **52**, 6704-6713 (2019).
2. Hu, Y. H., Zhao, X. H., Vlassak, J. J. & Suo, Z. G. Using indentation to characterize the poroelasticity of gels. *Appl. Phys. Lett.* **96**, 121904 (2010).
3. McKee, C. T., Last, J. A., Russell, P. & Murphy, C. J. Indentation versus tensile measurements of Young's modulus for soft biological tissues. *Tissue Eng. Part B Rev*. **17**, 155-164 (2011).
4. Hoshino, K. I., Nakajima, T., Matsuda, T., Sakai, T. & Gong, J. P. Network elasticity of a model hydrogel as a function of swelling ratio: from shrinking to extreme swelling states. *Soft Matter* **14**, 9693-9701 (2018).
5. Bokobza, L. Near infrared spectroscopy. *J. Near Infrared Spectrosc.* **6**, 3-17 (1998).
6. Swinehart, D. The Beer-Lambert law. *J. Chem. Educ.* **39**, 333-335 (1962).
7. Matsuda, T., Kawakami, R., Nakajima, T. & Gong, J. P. Crack tip field of a double-network gel: visualization of covalent bond scission through mechanoradical polymerization. *Macromolecules* **53**, 8787-8795 (2020).
8. Lake, G. J. & Thomas, A. G. . *Proc. R. Soc. Lond. A* **300**, 108-119 (1967).
9. Rubinstein, M. *Polymer Physics* Ch. 7 (Oxford Univ. Press, 2003).
10. Matsuda, T., Kawakami, R., Namba, R., Nakajima, T. & Gong, J. P. Mechanoresponsive self-growing hydrogels inspired by muscle training. *Science* **363**, 504-508 (2019).
11. Matsuda, T. Study on the self-growing materials in response to mechanical stimuli using double-network system. PhD dissertation (Hokkaido Univ. 2019).
