# Supplementary material for: Force-triggered rapid microstructure growth on hydrogel surface for on-demand functions
Source: Nat Commun. 2022 Oct 20;13:6213. doi: 10.1038/s41467-022-34044-8 (PMC9585076; doi:10.1038/s41467-022-34044-8)
Supplement: Supplementary file 3 — Description of Additional Supplementary Files [file 41467_2022_34044_MOESM3_ESM.pdf]

File Name: Supplementary Movie 1

Description: Double-network (DN) hydrogel was immersed in a concentrated *N*-isopropylacrylamide (NIPAm) aqueous solution and pressed with a macro-size indenter of 4 mm-diameter. Upon pressing the indenter, the transparent hydrogel rapidly turns to turbid in the pressed region within seconds. The results indicate that force-triggered polymer strand scission induces rapid radical polymerisation of NIPAm monomer to form poly(*N*-isopropylacrylamide) (PNIPAm).
