# Supplementary material for: Force-triggered rapid microstructure growth on hydrogel surface for on-demand functions
Source: Nat Commun. 2022 Oct 20;13:6213. doi: 10.1038/s41467-022-34044-8 (PMC9585076; doi:10.1038/s41467-022-34044-8)
Supplement: Supplementary file 5 — Reporting Summary [file 41467_2022_34044_MOESM5_ESM.pdf]

nature portfolio

Corresponding author(s): Dr. Tasuku Nakajima, Dr. Jian Ping Gong

Last updated by author(s): Oct 2, 2022

Reporting Summary

Nature Portfolio wishes to improve the reproducibility of the work that we publish. This form provides structure for consistency and transparency in reporting. For further information on Nature Portfolio policies, see our Editorial Policies and the Editorial Policy Checklist.

Statistics

For all statistical analyses, confirm that the following items are present in the figure legend, table legend, main text, or Methods section.

n/a	Confirmed	
☐	☒	The exact sample size (n) for each experimental group/condition, given as a discrete number and unit of measurement
☐	☒	A statement on whether measurements were taken from distinct samples or whether the same sample was measured repeatedly
☒	☐	The statistical test(s) used AND whether they are one- or two-sided<br>*Only common tests should be described solely by name; describe more complex techniques in the Methods section.*
☒	☐	A description of all covariates tested
☒	☐	A description of any assumptions or corrections, such as tests of normality and adjustment for multiple comparisons
☐	☒	A full description of the statistical parameters including central tendency (e.g. means) or other basic estimates (e.g. regression coefficient) AND variation (e.g. standard deviation) or associated estimates of uncertainty (e.g. confidence intervals)
☒	☐	For null hypothesis testing, the test statistic (e.g. F, t, r) with confidence intervals, effect sizes, degrees of freedom and P value noted<br>*Give P values as exact values whenever suitable.*
☒	☐	For Bayesian analysis, information on the choice of priors and Markov chain Monte Carlo settings
☒	☐	For hierarchical and complex designs, identification of the appropriate level for tests and full reporting of outcomes
☒	☐	Estimates of effect sizes (e.g. Cohen's d, Pearson's r), indicating how they were calculated

Our web collection on statistics for biologists contains articles on many of the points above.

Software and code

Policy information about availability of computer code

Data collection	Software VK Analyzer(v.2.4; Keyence Inc.), Software Image J (win-Java 8), Software Spectra Manager (FT/IR-6600)
Data analysis	Software VK Analyzer(v.2.4; Keyence Inc.), GraphPad Prism 8 software (v.8.0.2; GraphPad Software Inc.), Origin Pro 8.5 software (v. 8.5.1; OriginLab Inc.)

For manuscripts utilizing custom algorithms or software that are central to the research but not yet described in published literature, software must be made available to editors and reviewers. We strongly encourage code deposition in a community repository (e.g. GitHub). See the Nature Portfolio guidelines for submitting code & software for further information.

Data

Policy information about availability of data

All manuscripts must include a data availability statement. This statement should provide the following information, where applicable:

- Accession codes, unique identifiers, or web links for publicly available datasets
- A description of any restrictions on data availability
- For clinical datasets or third party data, please ensure that the statement adheres to our policy

The authors declare that data supporting the findings of this study are available within the paper and its supplementary information files. Source data is provided with this paper, which is available for Figures 2c,2d, 3a, 3b, 3c, 3d, and 6d.

Human research participants

Policy information about studies involving human research participants and Sex and Gender in Research.

Reporting on sex and gender	not applicable
Population characteristics	not applicable
Recruitment	not applicable
Ethics oversight	not applicable

Note that full information on the approval of the study protocol must also be provided in the manuscript.

Field-specific reporting

Please select the one below that is the best fit for your research. If you are not sure, read the appropriate sections before making your selection.

☒ Life sciences ☐ Behavioural & social sciences ☐ Ecological, evolutionary & environmental sciences

For a reference copy of the document with all sections, see nature.com/documents/nr-reporting-summary-flat.pdf

Life sciences study design

All studies must disclose on these points even when the disclosure is negative.

Sample size	The microstructures sizes were calculated by measuring the contour lines of microstructures using a three-dimensional microscope, as indicated in figure 2 legends. The platform area in the microstructure contour lines represents the true height value (figure 2b), which is verified by the microscopic image (figure 2a). Larger peaks at the edges of the contour lines may come from micro-cracks caused by stress concentration. We verified that large peaks at the edges don't represent the true microstructure height based on multiple control experiments. In subsequent comparison experiments, we compared the effect on microstructures from different parameters and monomers based on consistent criteria (figure 2c,d; figure 3c). There are many small peaks on the contour lines, and the fluctuation range of these peaks is very small (<0.9 µm), which has negligible effect on the calculation of the microstructure height. The Error bars in the figures represent the standard deviation from three replicates. Data in all figures are presented as mean values +/- standard deviations (SD). For the controlled water transportation experiments, the values of three replicates on three samples were averaged for each tested condition.
Data exclusions	No data was excluded from the analysis.
Replication	Data were pooled from three independent experiments for the following figures: Fig. 1c-d, Fig. 2c-d, Fig. 3a,c-d, Fig. 4a-c, Fig. 5a,b, and Fig. 6a-d. The results of these experiments were reproducible.
Randomization	The samples/organisms/participants allocation were random.
Blinding	The investigators were blinded to group allocation during the data collection and /or analysis.

Reporting for specific materials, systems and methods

We require information from authors about some types of materials, experimental systems and methods used in many studies. Here, indicate whether each material, system or method listed is relevant to your study. If you are not sure if a list item applies to your research, read the appropriate section before selecting a response.

Materials & experimental systems

n/a	Involved in the study	
☒	☐	Antibodies
☐	☒	Eukaryotic cell lines
☒	☐	Palaeontology and archaeology
☒	☐	Animals and other organisms
☒	☐	Clinical data
☒	☐	Dual use research of concern

Methods

n/a	Involved in the study	
☒	☐	ChIP-seq
☒	☐	Flow cytometry
☒	☐	MRI-based neuroimaging

Eukaryotic cell lines

Policy information about cell lines and Sex and Gender in Research

Cell line source(s)	The mouse myoblast cell line (C2C12) labelled with green fluorescent protein (GFP) purchased from RIKEN Cell Bank was used in the research.
Authentication	None of the cell lines used were authenticated.
Mycoplasma contamination	The cell lines were not tested for mycoplasma contamination.
Commonly misidentified lines (See ICLAC register)	N/A
